# Biotechnological approaches to creation of hypoxia and anoxia tolerant plants

**Published:** 2014

**Authors:** B. B. Vartapetian, Y. I. Dolgikh, L. I. Polyakova, N. V. Chichkova, A. B. Vartapetian

**Affiliations:** Timiryazev Institute of Plant Physiology, Russian Academy of Sciences, Botanicheskaya Str., 35, Moscow, Russia, 127276; A.N.Belozersky Institute of Physico-Chemical Biology Moscow State University

**Keywords:** anaerobic stress, growth index, in vitro cell selection, programmed cell death, transgenic plants, mitochondrial ultrastructure

## Abstract

The present work provides results of a number of biotechnological studies aimed at creating cell lines and entire plants resistant to anaerobic stress. Developed biotechnological approaches were based on earlier fundamental researches into anaerobic stress in plants, so “Introduction” briefly covers the importance of the problem and focuses on works considering two main strategies of plants adaptation to anaerobic stress. Those are adaptation at molecular level where key factor is anaerobic metabolism of energy (true tolerance) and adaptation of the entire plant via formation of aerenchyma and facilitated transportation of oxygen (apparent tolerance). Thus, sugarcane and wheat cells resistant to anaerobic stress were obtained through consecutive *in vitro* selection under conditions of anoxia and absence of exogenous carbohydrates. Tolerant wheat cells were used to regenerate entire plants of higher resistance to root anaerobiosis. It has been demonstrated that cells tolerance to anoxia is significantly supported by their ability to utilize exogenous nitrate. Cells tolerance established itself at the genetic level and was inherited by further generations. Apart from that, other successful attempts to increase tolerance of plants to anaerobic stress by means of stimulation of glycolysis and overexpression of genes responsible for cytokinin synthesis and programmed cell death are also discussed. The presented data proved the notion of two main strategies of plants adaptation to anaerobic stress proposed earlier on the base of fundamental studies.

## INTRODUCTION

As plants are obligate aerobes, oxygen deficiency (hypoxia) and especially its total absence (anoxia) cause dramatic ecological stress. Meanwhile plants often suffer from sudden molecular oxygen deprivation both under natural condition and as a result of human activity. Most often plants are subject to oxygen deprivation on hydromorphic and flooded soils for the oxygen’s poor solubility and low diffusion rate in the water [1, 2]. Nowadays, there are vast areas of hydromorphic soils in many countries [3-5]. It is believed that melting of permafrost and polar ice, together with ensuing rise of the world ocean level, may lead to a flooding of numerous regions of the planet. Oxygen scarcity is also observed in firm soils [6]. In this respect, roots and seeds of plants are the most vulnerable. In northern countries and countries with a moderate climate winter cereals and perennials can be damaged by ice crust, impermeable to gas, that appears on the surface of soil in autumn and winter [7]. Anaerobic stress may damage and even lead to a total failure of crop and wildings thus causing considerable ecologic and economic losses.

Problem of hypoxia and anoxia is also important in regard to long-term storage of agricultural commodities like fruits, grain, vegetables [8].

In last decades anaerobic stress in plants has become a topical subject of study not only among physiologists and biochemists, but molecular biologists and geneticists as well. Number of publications and ISPA conferences devoted to study of hypoxia and anoxia in plants is constantly rising. Anaerobic stress has been discussed in numerous special issues of international journals (see Annals of Botany (special section). 1994. V. 74. No 3. Ed. Jackson M.B.; Annals of Botany (special Issue). 1997. V. 79. Ed. Jackson M.B.; Annals of Botany (special section). 2002. V. 90. No 4. Ed. Smirnov N.; Annals of Botany (special Issue). 2003. V. 91. Eds Visser E., Voesenek L.A.C.J., Jackson M.B.; Russian J. of Plant Physiology (Special Issue). 2003. T. 50. No. 6. Ed. by Vartapetyan B. B. ; Annals of Botany (special Issue). 2005. V. 96. Ed. Jackson M.B.; Annals of Botany (special Issue). 2009. Eds Jackson M.B., Ishizawa K., Ito O.; New Phytologist (special Issue). 2011. V. 190. № 2. Eds Perata P., Armstrong W., Voesenek L.A.C.J.) Along with that a number of monographs [9-14] issued by ISPA members have had a significant impact on further development of this scientific trend.

Commonly admitted notion of two main strategies of plants adaptation to anaerobic stress has been actively elaborated. The first one is molecular adaptation which takes place at the absence or lack of oxygen through fundamental rearrangement of the entire cell metabolism. The second is adaptation of the plant as an entire body due to transportation of oxygen from aerial parts to parts localized in anoxic environment (roots), that is escape strategy to avoid anaerobiosis. It becomes more and more clear that cell energy metabolism is a key factor in both metabolic adaptation and plant damage under anaerobic stress.

High sensitivity of plants to the lack of oxygen, especially its total absence, can be explained by the fact that higher plants, being obligate aerobes, demand constantly available molecular oxygen in the environment to maintain itself.

However, many species, mainly wildings, in the course of evolution acquired the ability to inhabit temporarily or constantly hydromorphic and even flooded anaerobic soils [15, 16]. The only exception among cultivated plants is rice – *Oryza sativa *L*. *– that is known to be grown mostly on flooded soils [17-19]. Nevertheless, it is often that even rice plants suffer from anaerobic stress when sprouts get entirely submerged as it happens in monsoon season in East and South-East Asia [20].

The fact that numerous plants in the course of natural evolution or due to man-made selection acquired an ability to inhabit temporarily or constantly flooded anaerobic soils makes it important and viable to carry out both fundamental studies having their aim in finding out molecular mechanisms of plants adaptation and applied ones in particular biotechnological approaches (gene and cell engineering) to creation of plants tolerant to anaerobic stress. However, researches in this field were mostly fundamental and they prepared serious base for more active studies of applied problems of anaerobiosis, in particular development of biotechnological methods of creation plants tolerant to hypoxia and anoxia, which was the main objective of this review.

The present review discusses biotechnological approaches elaborated on the base of earlier fundamental studies in this field, in particular on the notion of two strategies of plants adaptation to anaerobic stress [15-17, 21, 22]. One of these approaches was devised from results of studies that demonstrated key role of anaerobic metabolism (glycolysis and fermentation) and carbohydrates metabolism in adaptation of plants to anaerobic stress [21-24]. This knowledge allowed to create cell lines of *Saccharum officinarum *L. [22, 25] and *Triticum aestivum *L*. *[26, 27] via *in vitro *selection in the absence of exogenous carbohydrates, that were more resistant to anoxia than original callus cells. More resistant cells of *T. aestivum *L. were further used to regenerate entire plants that occurred to be more tolerant to the soil anaerobiosis as their cultivation in ample water conditions proved.

Experimental results on *in vitro *selection of plant cells showing protective function of exogenous nitrate as potential alternative acceptor of electrons in severe conditions of anoxia have been also reviewed [28].

Taking into account the role of energy metabolism in adaptation of plants to anaerobic stress the possibility of increasing plants resistance via super expression of PDC and ADH genes were considered [29-32].

Another biotechnological approach, also successfully applied to obtain plants resistant to hydromorphic soils [33], was absolutely different from already mentioned. In this case another widely-known fact was used, namely the reaction of majority plants sensitive to hypoxia and anoxia to anaerobic stress: flooding at an early stages lead to withering and aging of aboveground organs and only after that to death. On the other hand, it is well known that aging of plants is subject to hormonal regulation. Thus, cytokinines make substantially rejuvenate aging aerial organs [34, 35]. Considering these circumstances, there was an attempt to enhance capability of plants to synthesize cytokinin and thus to boost their resistance to anaerobic stress by means of transformation of the plant with *ipt *gene responsible for the synthesis of this hormone [26, 33].

Possibility to increase plants resistance to anaerobic stress was also studied in transgenic tobacco *N. tabacum* Samsun NN characterized by high activity of recently discovered enzyme phytaspase [36-38] mediating programmed cell death. An interest to transgenic tobacco derives from earlier studies [15, 17, 22] that demonstrated not the molecular metabolic adaptation but crucially different pattern of plant adaptation to anaerobic environment expressing itself in formation of aerenchyma and avoidance of anaerobiosis due to longdistance transportation of oxygen that is the adaptation on the level of the entire plant body.

## IN VITRO SELECTION OF SACCHARUM OFFICINARUM CELLS TOLERANT TO ANAEROBIC STRESS

**Fig. 1:**
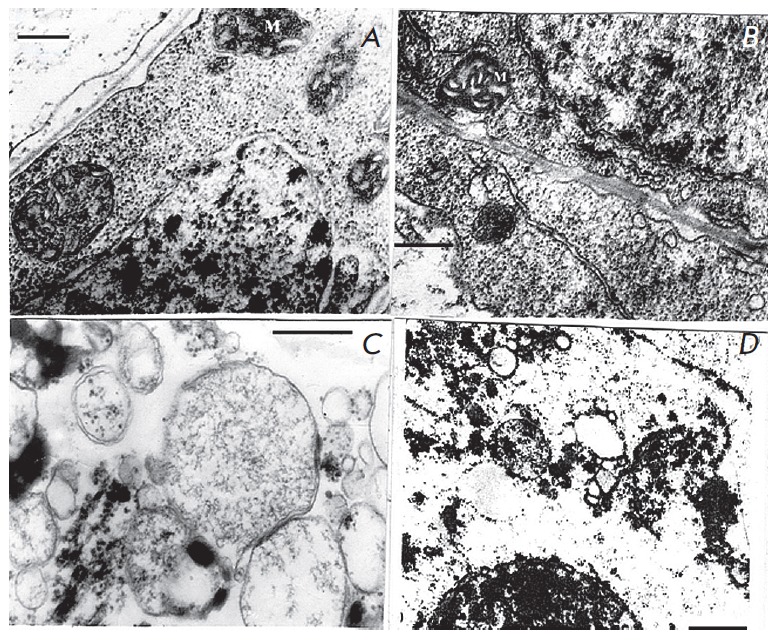
Ultrastructure of *Saccharum officinarum L *callus cells sensitive to anaerobic stress.Under anoxia and in the absence of exogenous glucose. a – control; b – 24 h anaerobic incubation; c – 48 h anaerobic incubation; d – 72 h anaerobic incubation. M – mitochondria Bars = 0.5μm

**Fig. 2:**
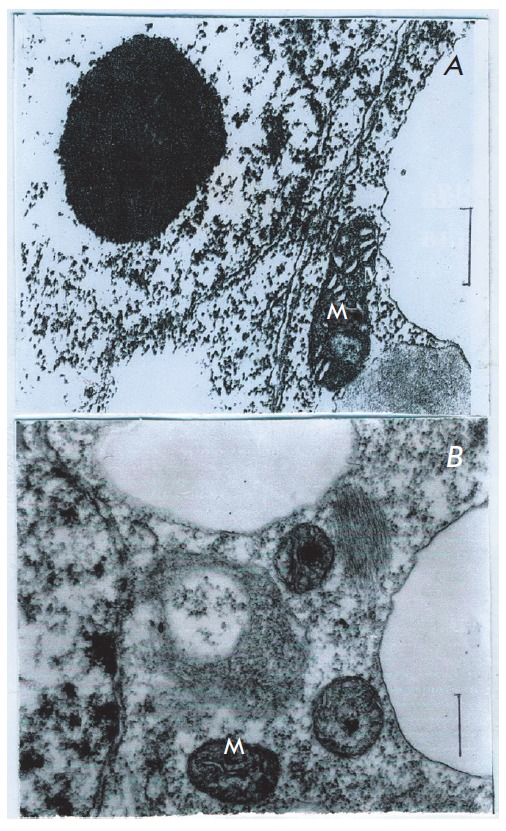
Ultrastructure of *Saccharum officinarum L* of callus cells sensitive to anaerobic stress. Under condition of anoxia and in the presence of 3% glucose. a – control; b – 96 h anaerobic incubation. M – mitochondria Bars = 0.5μm

As it has already been mentioned the basis for this biotechnological approach was experimental results proving the key role of anaerobic metabolism in resistance of various organs of plants to hypoxia and anoxia [15-17, 21-24]. Key role of anaerobic metabolism in resistance to anoxia and hypoxia is also confirmed by presented electron micrographs of ultrastructure of mitochondria from sugarcane callus cells that was transferred from aerobic medium to anaerobic in the absence and the presence of exogenous glucose (*Fig. 1* and *Fig. 2*). The stength of cells resistance to anaerobic stress was assessed through electron-microscopic study of ultrastructure of mitochondria that are highly sensitive to the lack of oxygen. Oxygen deprived mitochondria membranes demonstrate certain change pattern of ultrastructure.

In the absence of exogenous carbohydrates in the medium, ultrastructure of mitochondria and other organelles of cells, kept for 24 hours under anoxia (*Fig. 1B*), did not significantly differ from aerobic control (*Fig. 1A*). At the longer anaerobiosis (48 hours) more distinct features of destruction in mitochondria and other organelles were detected (*Fig. 1C*). Anoxia of 72 hour length caused total degradation of mitochondria and other cell organelles (*Fig. 1D*).

On the contrary, addition of glucose in concentration of 3% to a medium demonstrated no destructive changes of mitochondria and organelles even at anaerobiosis of 96 hours (*Fig. 2B*).

Alongside the electron microscopy observations the ability of callus to restore its growth after anaerobic period in conditions close to normal aeration was also assessed. To do this, we defined callus growth index (the difference between the final and the initial masses divided by the initial) following one month of cultivating in normal conditions.

Results of these experiments indicated the decrease of post-anaerobic growth index for cells in the absence of exogenous carbohydrates as the duration of anaerobiosis increases; at 96-hour anaerobiosis this index tends to zero (*Table 1*).

**Table 1:** Growth index of *Saccharinum officinarum L* exposed to varying in length anaerobic incubation on glucose-free medium after cultivation under normal aeration conditions for 1 month

Anaerobic incubation, h	Growth index	% of control
0	4.63 ± 0.50	100
6	2.35 ± 0.25	50.7 ± 5.4
24	1.47 ± 0.15	31.7 ± 3.2
48	0.60 ± 0.09	13.0 ± 2.0
72	0.55 ± 0.10	11.8 ± 2.2
96	0.16 ± 0.01	3.5 ± 0.32

Cells demonstrated substantially better resistance to anoxia at administration of exogenous glucose (*Table 2*).

**Table 2:** Growth index of *Saccharinum officinarum L* exposed to anaerobic incubation anaerobic incubation on medium containing 3% glucose after cultivation under normal aeration conditions for 1 month

Anaerobic incubation, days	Growth index	% of control
0	5.7 ± 0.51	100
3	3.0 ± 0.29	52.0 ± 5.0
5	2.9 ± 0.31	50.8 ± 5.4
7	2.5 ± 0.27	43.8 ± 4.7
9	1.5 ± 0.14	26.3 ± 2.4
14	0.2 ± 0.06	3.3 ± 1.1

Since the experiments with cells of sugarcane callus confirmed the key role of anaerobic energy metabolism in formation of cells resistance to anoxia, further selection of tolerant callus cells [25-28] and anaerobic exposure were carried out in the absence of exogenous carbohydrates (glucose) in the medium. Only in those conditions when cells resistance was defined by parameters of anaerobic energy metabolism of endogenous carbohydrates one could hope to obtain cells genuinely tolerant to anoxia.

**Fig. 3:**
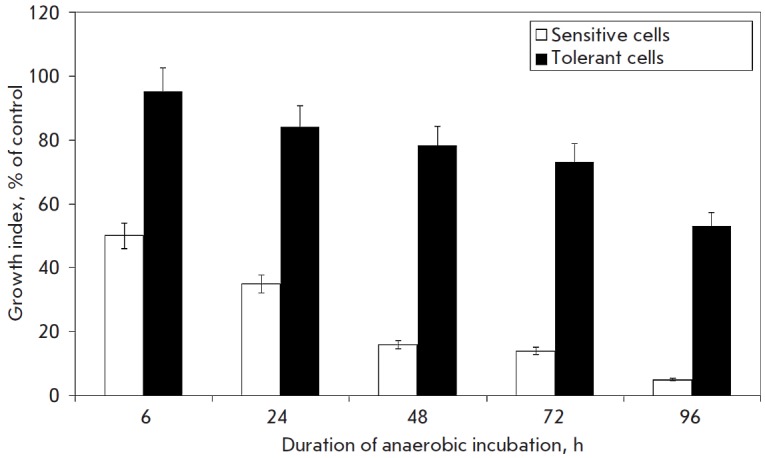
Growth index of* Saccharum officinarum L *sensitive and tolerant cells, that were, after anaerobic incubation, cultivated during one month under condition of normal aeration. White column – callus cells before *in vitro* selection. Shaded column – callus cells after *in vitro *selection

From the results of trial experiments a consecutive* in vitro *selection of anoxia-tolerant cells in carbohydrate- free medium was derived. After 48-hour incubation 13% of cells remained capable of growing further in aerobic conditions. Calluses were incubated without oxygen for 48 hours and kept under normal aeration. Clones, formed under anaerobic incubation (48 hours), were then exposed to anoxia for 72 hours. From cells that survived the second stage of selection and underwent subsequent anaerobiosis for 96 hours aerobically growing clones were picked. Thus after three stages of selection we obtained a cell line of sugarcane that grew in post-anaerobic period under conditions of normal aeration more actively than original callus. Even after exposure to anoxia for 96 hours half of such cells remained capable of dividing, whereas the original callus showed such capability only after 6-hour anaerobiosis (*Fig. 3*).

## IN VITRO SELECTION OF ANOXIA-TOLERANT WH EAT CELLS AND REGENERATION OF ENTIRE PLANTS RESISTANT TO FLOODING OF ROOTS

**Fig. 4:**
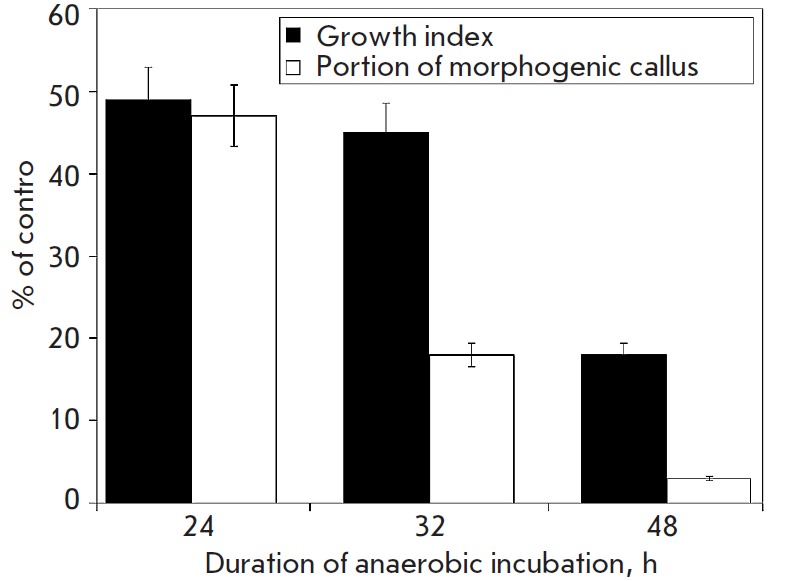
Growth index and percent of morphogenic calli of *Triticum aestivum L*. after anaerobic incubation in the absence of exogenous glucose. Control – under aerobic condition

Similar experiments on selection of tolerant cells in the absence of exogenous carbohydrates were carried out with *T. aestivum *L. callus in order to regenerate the entire plant, resistant to flooding of roots [27]. In the course of selection cells exposed to anoxia were gradually losing the ability either to grow further in aerobic conditions or to regenerate into new plants, so we selected cells under conditions of 32 hour anaerobiosis. In this case cell growth index comprised 45%, whereas the capability of regenerating a whole plants from tolerant cells – 18% (*Fig. 4*).

Plants obtained from tolerant cells after selection under anoxia were tested together with controls for flooding of roots during 16 days at the temperature of 26°C. Among controls only 30% of plants survived, whereas among regenerants – about 73%.

In order to find out the genetic aspects of tolerance to soil anaerobiosis we sowed seeds acquired from regenerated tolerant plants of the first generation and then assessed tolerance of new plants to root anaerobiosis. R1 plants obtained as a result of self-pollination of regenerant plants were examined for root flooding in soil experiment at the average temperature of 32 and 22°C *(Table 3).* At all used temperatures survival rate among descendants of regenerated plants was higher than that among controls.

**Table 3:** Survival of *Triticum aestivum L plants* R_1_ and R_2_ under conditions of root anaerobiosis at different temperature regimens

Experimental conditions	Plants	Total number of plants	Survived plants	
			N	Rate, %
8 day flooding, 32°C	Control R_1_	20 22	0 7	0 32
10 day flooding, 22°C	Control R_1_	32 36	18 36	56 100
8 day flooding, 32°C	Control R_2_	24 32	11 32	46 100

**Fig. 5:**
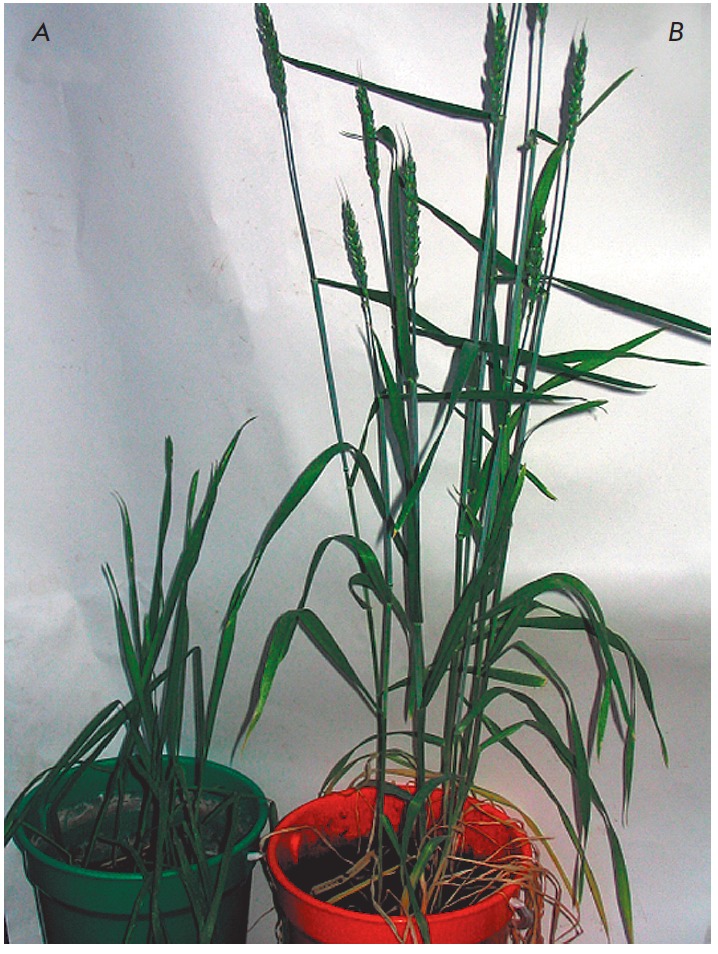
Wheat plants after 8-days flooding. Control (A) and tolerant (B) plants

In the soil experiment R2 plants remained tolerant to the root flooding (*Table 3*, *Fig. 5*). Thus it was confirmed that regenerant plants inherit increased tolerance to flooding.

## PROTECTIVE ROLE OF NITRATE IN ANOXIA TOLERANCE OF S. OFFICINARUM CELLS OBTAINED THROUGH IN VITRO SELECTION

Further experiments on anoxia tolerant cells of *S. officinarum*, isolated in the course of selection, and callus cells as control were an attempt to find out possible role of exogenous nitrate (NO_3_^-^) as protective factor at anaerobic incubation of cells [28]. Previous studies carried out on whole plants and separate organs showed mobilization and utilization of exogenous nitrate to play significant role in plants tolerance at the absence of molecular oxygen [39-44].

**Fig. 6:**
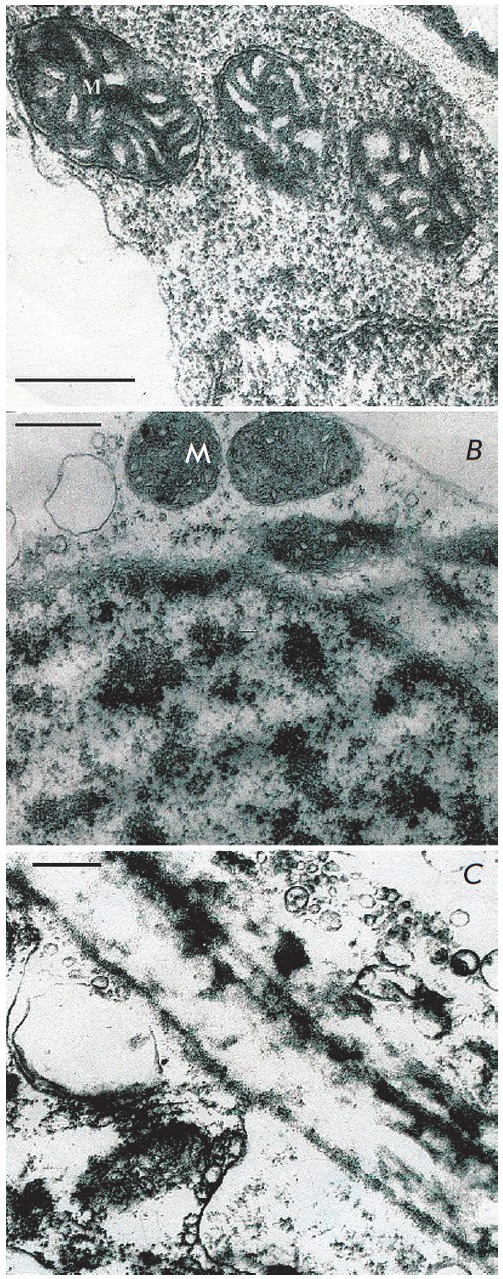
Ultrastructure of *Saccharum officinarum L. *sensitive callus cells under conditions of anoxia and in the absence of exogenous nitrate. a – control; b – 6 h anaerobic incubation in the absence of exogenous nitrate; c - 24 h anaerobic incubation in the absence of nitrate. M – mitochondria Bars = 0.5μm

Electron microscopic examination of cells of original sugarcane callus (control) showed their high sensitivity to anoxia in the absence of nitrate in the medium (*Fig. 6*). Although 6-hour anaerobic incubation did not cause any serious damage to mitochondria membranes, following 24-hours of anaerobiosis we observed not only damaged membranes, but entirely degraded both mitochondria and other cell structures (*Fig. 6C*).

**Fig. 7:**
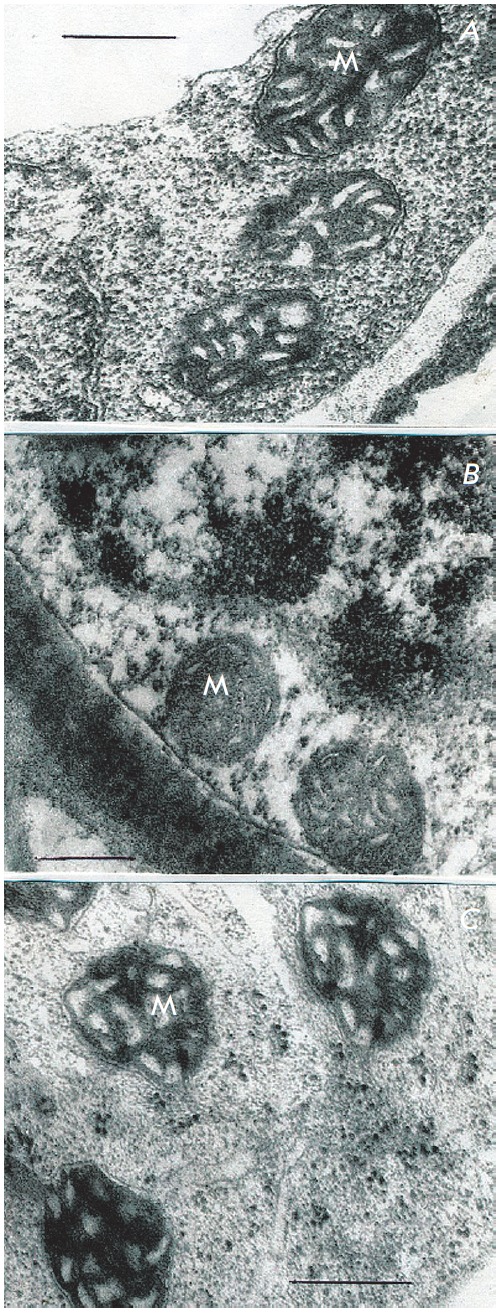
Ultrastructure of *Saccharum officinarum L. *t sensitive callus cells under conditions of anoxia and in the presence of exogenous nitrate. a – control; b – 6 h anaerobic incubation; c – 24 h anaerobic incubation. M – mitochondria Bars = 0.5μm

In the presence of exogenous nitrate callus cells of the control intolerant line were demonstrated to have increased anoxia tolerance. Even at the 24-hour anaerobiosis were detected no obvious signs of membrane degradation in mitochondria and other organelles (*Fig. 7C).* Substantial destruction of mitochondria membranes close to degradation was observed at longer anaerobiosis (48 hours).

**Fig. 8:**
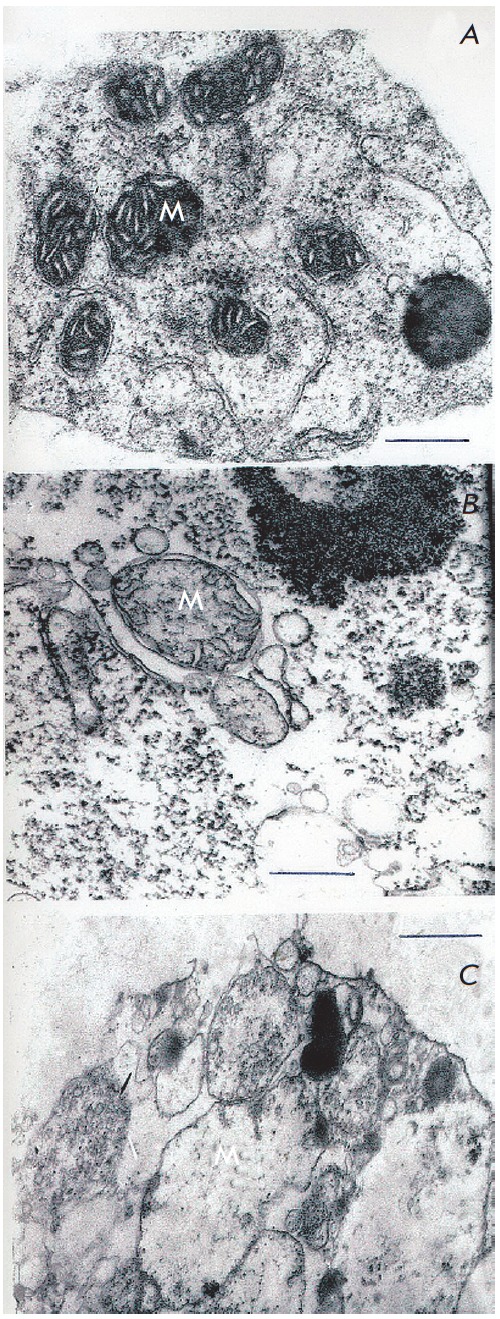
Ultrastructure of *Saccharum officinarum L. *tolerant callus cells under conditions of anoxia and in the absence of exogenous nitrate a – control; b – 24 h anaerobic incubation; c – 48 h anaerobic incubation M – mitochondria Bars = 0.5μm

Cells of tolerant line obtained through *in vitro *selection even in the absence of nitrate in the medium were significantly more resistant to anoxia than original cells *(Fig. 8).* Anaerobic incubation of cells during 24 hours did not cause destruction of membranes (*Fig. 8B*). Only after 48 hours of anaerobiosis evident signs of mitochondrial destruction were recorded (*Fig. 8C*) and only after 72 hours when the cells ultrastructure completely degenerated.

**Fig. 9:**
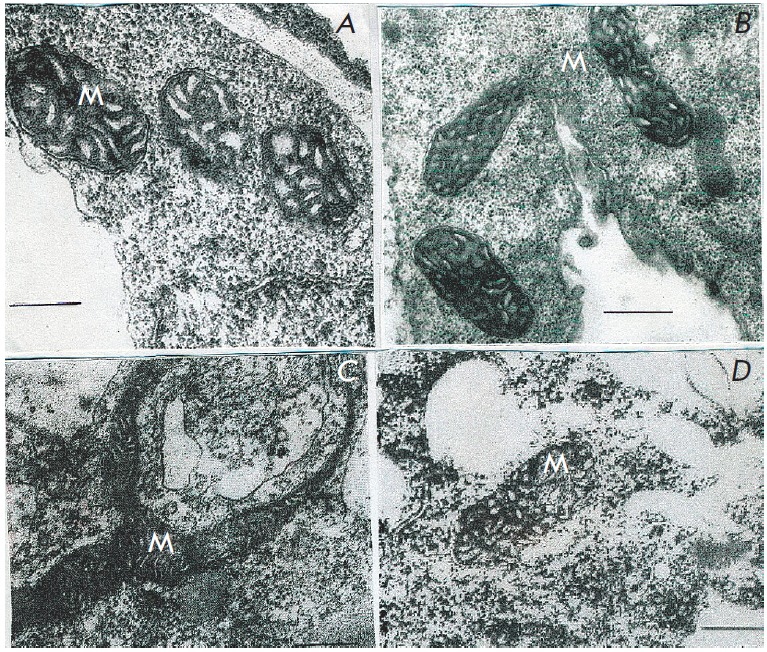
Ultrastructure of *Saccharum officinarum L. *tolerant callus cells under conditions of anoxia and in the presence of exogenous nitrate. a– 24 h anaerobic incubation; b, c – 48 h anaerobic incubation; d – 72 h anaerobic incubation. M – mitochondria Bars = 0.5μm

The most serious distinctions between callus lines were revealed at the anaerobic incubation of tolerant cells isolated through *in vitro *selection in the presence of nitrates in the medium. Mitochondria ultrastructure of such cells remained intact even after 48–72 hours of anaerobiosis (*Fig. 9B, C, D*) except for small non-pathological morphologic changes. However, these changes in ultrastructure and morphology were not destructive even after 72 hour exposure (*Fig. 9D*).

**Fig. 10:**
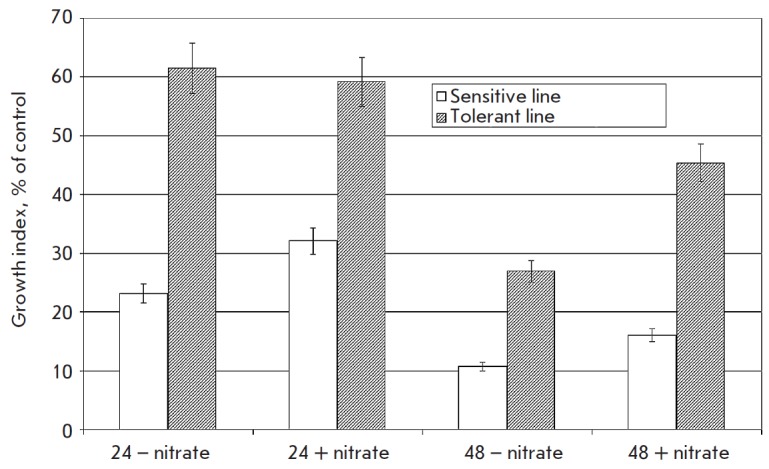
Growth index of* Saccharum officinarum L. *tolerant and sensitive cells after anaerobic incubation. The figures indicate the duration (h) of anaerobic incubation of callus cells in the presence and in the absence of nitrate Control – aerobic condition

Along with the monitoring of the cells ultrastructure under conditions of anaerobiosis in the presence and in the absence of nitrate we also monitored growth of cells from the sensitive and the resistant lines of callus in post anaerobic period. In the mentioned period growth of the sensitive cells in the nitrate-free medium was considerably suppressed. Supplement of nitrate did not make callus of the sensitive line to grow much better. For instance, following 48-hour of anoxia accretion comprised only 10% of the control level for nitrate-free medium and 16% for the full medium. Adding nitrate to the medium significantly favors growth of tolerant cells. Thus, after 48 hours of anaerobiosis callus mass grew 18% bigger in the nitrate-containing media than in the nitrate-free medium (*Fig. 10*). In the presence of nitrates tolerant cells remained able to grow even after 72 hours of anoxia, whereas in the nitrate-free medium such growth was not detected.

As far as calluses of the sensitive line are concerned, their exposure to anoxia stipulated considerable decrease of the growth index and compared to resistant line protective role of nitrate was expressed significantly weaker.

Thus, the gained results clearly indicate that under conditions of anoxia, exogenous nitrate serves a protective factor both in control cells and in cells obtained via* in vitro *selection in the absence of molecular oxygen. However, in cells acquired through *in vitro *selection protective function of exogenous nitrate was considerably stronger than that in original (control) cells.

Recent publications confirming positive influence of nitrate on plants exposed to hypoxia and anoxia showed similar results in the absence of nitrate, but in the presence of trace amount of nitrite in the medium [45-47]. These results let us assume that protective action of nitrate in the absence of oxygen is stipulated by electron-acceptor function of not nitrate, but, more likely, that of nitrite or by signal function of NO_2_^–^ that is formed from nitrate under anaerobiosis. On the other hand, as it was demonstrated in Hill laboratory [47] under conditions of hypoxia, as a result of NO_2_^–^ reduction, mitochondria synthesize ATP, likely one of the most important protective factors of both nitrite and nitrate, the latter being a source of nitrite under conditions of hypoxia and anoxia.

One should pay attention to the following trait of tolerant cells obtained through consecutive *in vitro *selection under anoxia stress: in the presence of nitrate anoxia tolerance of selected cells is significantly higher than that of original cells used for selection [28]. This observation entitles us to assume that anoxia tolerance of *S. officinarum *cells substantially rose in the course of selection defined by glycolytic reactions along with processes of nitrate and, probably, nitrite utilization, under such harsh conditions.

It appears viable to devote further attempts to finding out physiological function of nitrite formed from nitrate as potential alternative electron acceptor or signal factor in the course of anaerobic incubation of callus cells.

## ATTEMPTS TO BOOST THE RESISTANCE OF TRANSGENIC PLANTS TO ANAEROBIC STRESS BY MEANS OF STIMULATING THE ACTIVITY OF GLYCOLYTIC ENZYMES (PDC AND AD H)

On the assumption that anaerobic energy metabolism is a key factor in metabolic acclimation of plants to anaerobic stress [15, 21, 22, 48-53], there were numerous attempts to raise plants tolerance to hypoxia and anoxia by enhancing alcoholic fermentation with overexpression of genes of glycolytic enzymes in transgenic plants [29-32]. The results of the first experiments were somewhat controversial [29-31]. Experiments on the activity of alcoholic fermentation enzymes (PDC and ADH) in transgenic *Arabidopsis* [32] appeared especially interesting. Compared to tobacco roots [30], transgenic *Arabidopsis *with introduced PDC construct exhibited not only higher speed of alcoholic fermentation, but also greater resistance to hypoxia than control plants [32]. Unlike PDC transgenic* Arabidopsi*s, plants with ADH transgene and, respectively, higher ADH activity, did not demonstrate the increase in tolerance, although mutation in* adh1 *gene substantially enhanced accumulation of acetaldehyde and dramatically reduced the resistance to the hypoxic stress. High sensitivity and vulnerability of ADH mutant of *Arabidopsis *under hypoxia stress could be stipulated by accumulation of acetaldehyde, amount of which sharply rose in ADH deprived cells, and, consequently, by possibility of reduction of acetaldehyde to ethanol and thus protection of cells of its toxic effect. Along with that one cannot totally neglect the accumulation of pyruvic acid in these conditions that may to some extent lead to LDH-mediated accumulation of lactate in toxic concentrations. By administration of 3% sucrose authors demonstrated that the increase of resistance requires plants to be provided with substrate. This conclusion correlates the results of our earlier experiments [21] and works of other authors [48-57]. On the base of mentioned studies [32] it was concluded that PDC activity closely associated with the intensity of carbon flow in alcoholic fermentation and defines tolerance to hypoxia stress, i.e. PDC directly regulate alcoholc fermentation. Thus, results of *Arabidopsis *experiments [32] also confirm the idea that energy metabolism is a key factor to the true resistance of plant cells to anaerobic stress.

## ROOT FLOODING RESISTANCE OF TRANSGENIC PLANTS EXP RESS ING THE AGROBACTERIUM IPT GENE

Alongside finding means to increase the tolerance of wheat to anaerobic stress by *in vitro *cell selection in the absence of exogenous sugars and oxygen there was an attempt to obtain wheat plants more tolerant to root flooding by introducing isopentenyltransferase gene (*ipt), *coding key enzyme of cytokinin biosynthesis pathway [26]. The interest to stimulation of cytokinnin synthesis under anaerobic stress was dictated by the fact that cytokinin significantly contributes to preventing plants aging [34, 35]. It is commonly known that aboveground organs of oxygen-deprived plants on flooded soils are characterized by signs of premature aging like chlorosis, leaf fall and lesions [58, 59]. That is why there were attempts to slow down aging of transgenic *Arabidopsis* and wheat by stimulating cytokinin synthesis and thus to improve tolerance to anaerobic stress [26, 33, 60]. Level of isopenteniladenin recorded in transgenic wheat obtained in our experiments in conditions of flooding of roots was 30 times higher than that in untransformed plants. The impact of hormonal balance under anaerobic stress in transgenic and control plants was observed during the entire ontogenesis [26, 33].

In wheat experiments tolerant criteria were growth of the above-ground mass of transgenic and control plants and grain harvest under conditions when the root zone of plants had been flooded for 14 days *(Table 4)*. As it can be seen from the presented data, growth of the above-ground organs in control plants was considerably slower than that in transgenic plants with introduced *ipt *gene. Difference between the crop yield in experimental and control plants was even more striking. Yield was defined as the weight of crop (in gramms) harvested from 1 m^2^ of soil (g/m^2^).

**Table 4:** Change of linear dimensions and yield of ipttransgenic and control wheat plants exposed to 14 day root flooding (in relation to unflooded plants)

Parameters, % of control	Control plants	Transgenic plants
Average plant height increment over 14 days of root flooding	37	51
Portion of heads with seeds	33	89
Average seed weight	26	46
Yield	2	36

Alongside the mentioned we also monitored the activity of antioxidant enzymes (superoxide dismutase and catalase) and accumulation of malondialdehyde in control plants and in plants with introduced Agrobacterium* ipt *gene*. *By the end of the flooding period the amount of malondialdehyde found in transgenic plants was 32% lower than that in controls. On the contrary, activity of superoxide dismutase and catalase in transgenic wheat remained high during the entire hypoxic period, whereas in controls it was falling from the sixth day of the root flooding. These data is evidence that transgenic plants under hypoxia suffered less stress than controls.

Similar to results of Arabidopsis experiments [60], provided data indicate the positive effect of stimulation of cytokinin synthesis under anaerobic stress.

## ON THE POSS IBLE ROLE OF APOPTOTIC PROTEASE (PH YTASP ASE) IN THE INCREASE OF TOBACCO PLANTS TOLERANCE TO ANAEROBIC STRESS

Programmed cell death play a critical role both in development of plants and their reaction to stress including defense against pathogenic agents [61-67]. As it has been mentioned earlier, one of the main strategies of plants adaptation to hypoxia and anoxia is to avoid anaerbiosis by formation of spaces in roots (aerenchyma) as a result of apoptosis of a certain part of cells. Aerenchyma substantially alleviates long-distance transportation of oxygen from above-ground organs of plant to roots and rootstocks resting in anaerobic environment. Thus, it allows plants to survive even on the flooded soils.

However, aerenchyma forms mainly in wild species inhabiting flooded anaerobic soils. As cultivated plants do not possess such advanced ability to form aerenchyma, anaerobiosis often damages and kills them. Hence, of particular interest is recently discovered apoptotic protease phytaspase [36, 68-70], involved into programmed cell death in plants, the very same process during which aerenchyma forms. That is why in present work we tried to find out with the help of plants transformed with phytaspase gene whether it is possible to use this enzyme for formation of aerenchyma and thus to increase tolerance to hypoxia and anoxia in those cultivated species that do not possess such ability or possess a weakly developed one.

**Fig. 11:**
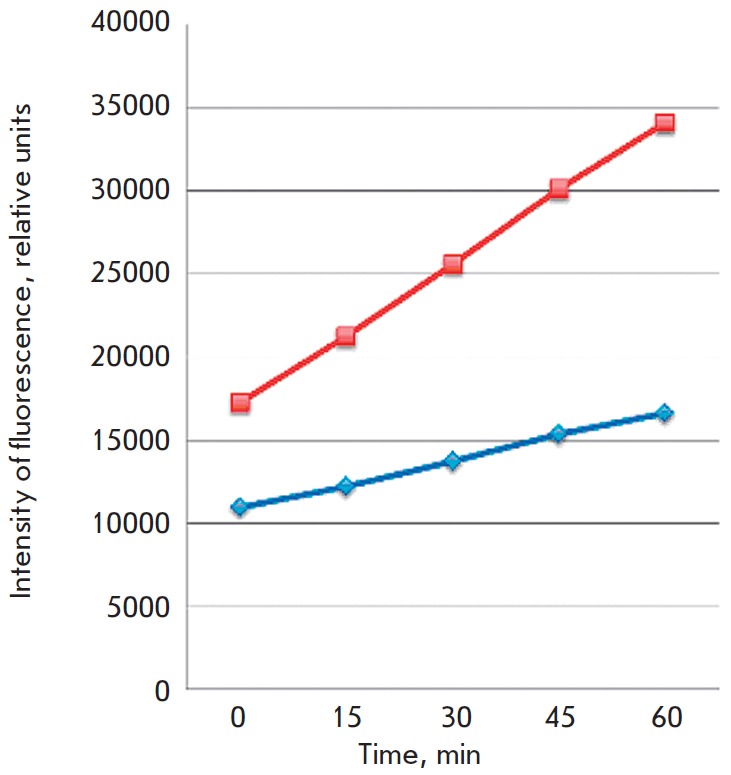
The rate of hydrolysis of phytaspase flurogenic peptid substrate (Ac-VEID – AFG) in tabaco leaves extracts .Transgenic (red) and wild (blue) plant tips

In order to do this we used transformed plants of* Nicotianum tabacum *expressing phytaspase gene and wild tobacco plants as control to compare their phenotypical and anatomical traits. Phytaspase activity in transgenic plants was 3 times higher than that in wild plants (*Fig. 11*).

**Fig. 12:**
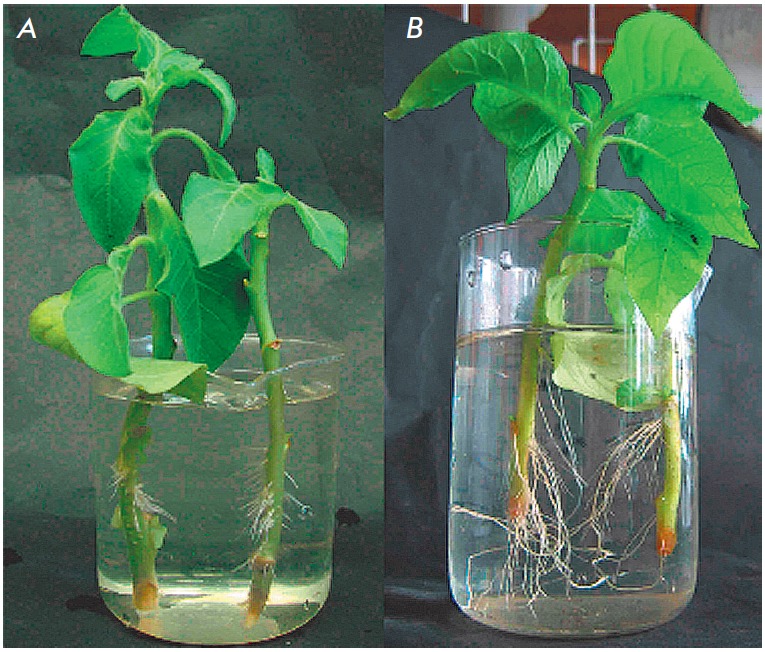
Rhizogenous of tabaco cuttings after 17 days of experiment starting. Wild (A) and transgenic (B) plants

**Fig. 13:**
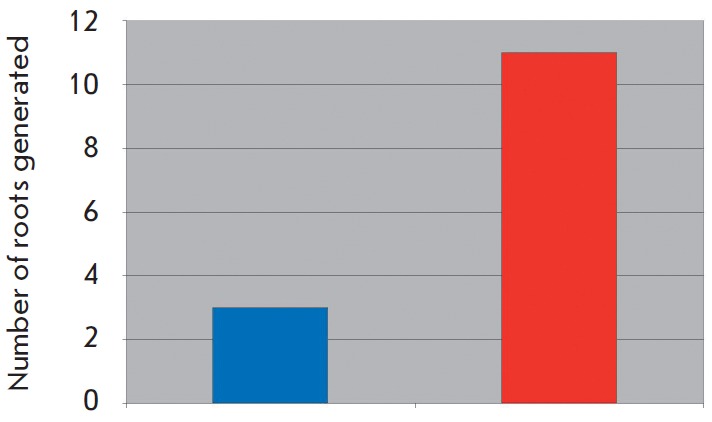
Rhizogenous of tabaco cuttings after 17 days of experiment starting. Wild (blue) and transgenic (red) plants

Results of trial experiments performed while transgenic and control plants were developing evidenced considerable differences even in conditions of normal aeration of the root zone and especially during rhisogenesis when shoots were put in water for 17 days (*Fig. 12, Fig. 13*). In transgenic plants the mentioned process went considerably active. The same concerned seed sprouting, growth of leaves and stems of young tobacco plants. All mentioned processes were twice active in transgenic plants.

Another question of our special interest was comparative analysis of anatomy of roots of transgenic and control plants as the enhanced activity of phytaspase, responsible for the programmed cell death was expected to contribute to formation of aerenchyma in roots of transgenic plants and to increase tolerance to the root anaerobiosis.

However, quantitave evaluation of intercellular spaces in the cross sections of roots of both transgenic plants and controls under normal aeration did not reveal significant differences.

As far as soil flooding is concerned, when roots suffer from anaerobic stress, intercellular spaces in transgenic plants were proved to be greater than those in controls by results of trial experiments (*Table 5*).

**Table 5:** Intercellular space area in the roots of transgenic and control plants under normal aeration and under root anaerobiosis

Experimental conditions	Intercellular space area / Total area of root core parenchyma	
	Control plants (wild type)	Transgenic plants
Normal aeration	3.53 ± 0.28	2.92 ± 0.68
Root anaerobiosis, 48h*	4.07 ± 1.1	11.45 ± 2.35

* The roots were flooded 5 cm above the soil surface

Consequently, results of trial experiments that require more thorough consideration and confirmation indicate that increased activity of phytaspase, involved into the programmed cell death, is favorable for formation of intercellular spaces (aerenchyma) in roots of transgenic plants under hypoxia.

## CONCLUSIONS

The present review is devoted to results of a number of experimental works in the course of which authors elaborated biotechnological approaches, including genetic engineering and methods of cell selection *in vitro*, to create plants tolerant to anaerobic stress. These approaches are based on earlier fundamental studies, in particular a notion on two main strategies of plants adaptation to hypoxia and anoxia: molecular acclimation where anaerobic energy metabolism of cells play major role (true tolerance) and adaptation of the whole plant by formation of aerenchyma and facilitated long-distance transportation of oxygen (apparent tolerance).

The notable contribution that was made into the creation of cells and plants tolerant to anaerobic stress in the considered works lets one hope that these results will serve a foundation of the new avenue of research in biotechnology and help the development of applied studies along with classical approaches to selection and hybridization.

It also should be mentioned that results of considered studies confirmed the idea of two main strategies of plants acclimation to hypoxia and anoxia and on key role of anaerobic energy metabolism in metabolic adaptation of plants to anaerobic stress that were previously suggested on basing on fundamental study of plants life under conditions of anaerobiosis.

## Abbreviations

PDC: pyruvate decarboxylase
ADH: alcohol dehydrogenase
ISPA: International Society for Plant Anaerobiosis
